# Automated classification of tertiary lymphoid structures in colorectal cancer using TLS-PAT artificial intelligence tool

**DOI:** 10.1038/s41598-025-94664-0

**Published:** 2025-03-21

**Authors:** Marion Le Rochais, Ikram Brahim, Rachid Zeghlache, Geoffroy Redoulez, Matthieu Guillard, Pierre Le Noac’h, Marine Castillon, Amélie Bourhis, Arnaud Uguen

**Affiliations:** 1 CHU de Brest, LBAI, UMR1227, Inserm, Univ Brest, 5, Avenue Foch, 29200 Brest, France; 2 CHU de Brest, LaTIM, UMR1101, Inserm, Univ Brest, Brest, France; 3 Pathology Department, CHU Brest, 29220 Brest, France; 4 Oncology Department, CHU Brest, 29220 Brest, France

**Keywords:** Colorectal cancer, Tertiary lymphoid structures, Immunohistochemistry, Artificial intelligence, Deep learning, QuPath, Cancer microenvironment, Colorectal cancer, Chronic inflammation, B cells, T cells, Inflammation, Lymphocytes, Lymphoid tissues, Tumour immunology

## Abstract

Colorectal cancer (CRC) ranks as the third most common and second deadliest cancer worldwide. The immune system, particularly tertiary lymphoid structures (TLS), significantly influences CRC progression and prognosis. TLS maturation, especially in the presence of germinal centers, correlates with improved patient outcomes; however, consistent and objective TLS assessment is hindered by varying histological definitions and limitations of traditional staining methods. This study involved 656 patients with colorectal adenocarcinoma from CHU Brest, France. We employed dual immunohistochemistry staining for CD21 and CD23 to classify TLS maturation stages in whole-slide images and implemented a fivefold cross-validation. Using ResNet50 and Vision Transformer models, we compared various aggregation methods, architectures, and pretraining techniques. Our automated system, TLS-PAT, achieved high accuracy (0.845) and robustness (kappa = 0.761) in classifying TLS maturation, particularly with the Vision Transformer pretrained on ImageNet using Max Confidence aggregation. This AI-driven approach offers a standardized method for automated TLS classification, complementing existing detection techniques. Our open-source tools are designed for easy integration with current methods, paving the way for further research in external datasets and other cancer types.

## Introduction

Recent data show that colorectal cancer (CRC) is the third most common and second deadliest cancer worldwide^1^. The immune system, particularly tertiary lymphoid structures (TLS), plays a key role in CRC prognosis^2,3^. TLS progress through distinct maturation stages from simple aggregates to structures with germinal centers (GC), reflecting evolving immune responses within the tumor microenvironment. The presence of mature GC-TLS has been associated with improved clinical outcomes and potentially better immunotherapy responses^3–5^, underscoring the importance of precisely characterizing these structural and functional transitions.

However, comparing TLS across studies is challenging due to variable histological definitions in H&E and IHC staining. While AI tools help assess TLS maturation in H&E slides^6,7^, H&E has poor sensitivity for mature TLS^8,9^. Immunohistochemistry (IHC) using CD21 and CD23 has proven effective for distinguishing TLS maturation stages, with CD21 positivity indicating the presence of FDCs and CD23 positivity signaling the formation of germinal centers^10^. Since IHC is routine in clinical practice, automating TLS quantification with CD21 and CD23 could offer a more efficient, objective, and reproducible method.

Deep learning could support automated TLS classification, addressing the increasing need for standardized TLS assessment in clinical trials^11,12^. This study presents an algorithm for automatic TLS classification in dual IHC-stained CRC slides using CD21 and CD23.In this study, we developed an algorithm to automatically classify TLS from conventional dual immunohistochemistry-stained whole-slide images (WSI) of CRC samples, using CD21-CD23 staining.

## Results

### Annotation methodology

**Fig. 1:**
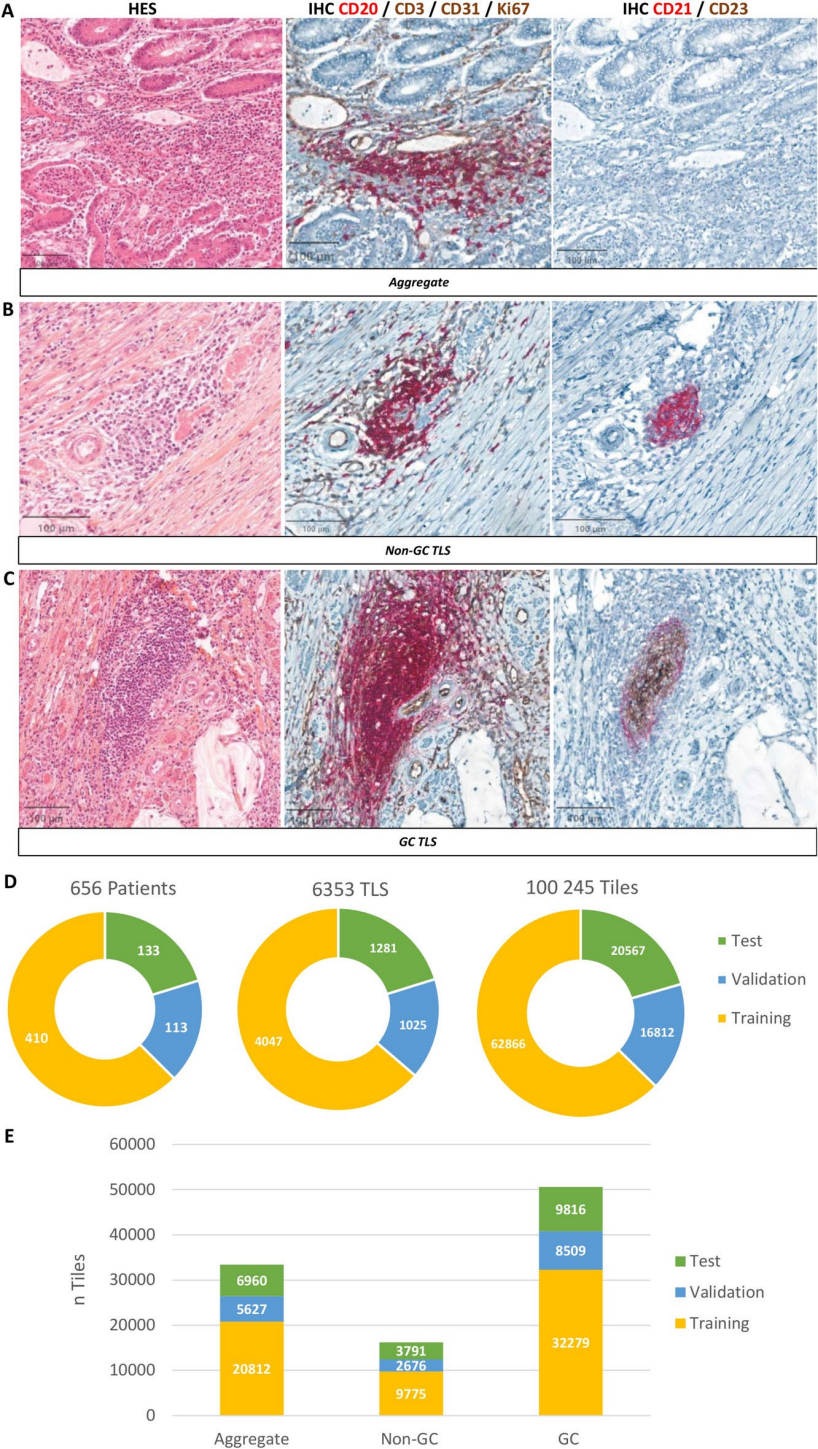
Annotations Methodology of the Dataset and Strategic Dataset Design for Balanced TLS Maturation Stage Classification. HES and IHC slides (CD20/CD3/Ki67/CD31) and CD21/CD23) for the characterization of the different TLS maturation class: Aggregate (**A**), Non-GC TLS (**B**) and GC TLS (**C**). The whole dataset was split into training, validation and test sets (**D**), with an equivalent repartition of each TLS maturation class (**E**).

To evaluate the maturation annotation reproducibility, three different raters (M.G, P.L.N, M.L.R) underwent independent training using a dataset comprising 9 typical cases, encompassing samples with no TLSs, aggregates, Non-GC TLS and GC TLS. For each case, raters learned how to assess the maturity status of TLSs based on CD21/CD23 IHC staining, categorized as 0 for Aggregate (Fig. 1A), 1 for Non-GC TLS (Fig. 1B), and 2 for GC-like TLS (Fig. 1C). Following this training phase, interrater agreement was evaluated using a cohort of 60 randomized CRC adenocarcinoma samples, and show that this method was reproducible and reliable with strong inter-rater level agreement (Cohen’s kappa value > 0.8) (see Supplementary data 1).

Then, using QuPath, the entire dataset was annotated by a single rater, encompassing a total of 656 WSI of 656 patients representing 6,353 TLS. From these, we extracted a total of 100,245 tiles (Fig. 1D). In mean, each TLS contained 15 tiles. The most prevalent class was GC TLS, with 50,604 tiles, followed by the Aggregate class with 33,399 tiles, and Non-GC TLS with 16,242 tiles (Fig. 1E). The dataset was then divided into training, validation, and test sets (Fig. 1D, E). The Training Set was composed of 410 patients and was utilized to train the models, allowing them to learn the intricate patterns and nuances present in the WSIs. The Validation Set consisted of 113 patients and was used to validate the models’ performance and fine-tune their parameters during the training phase.

**Fig. 2:**
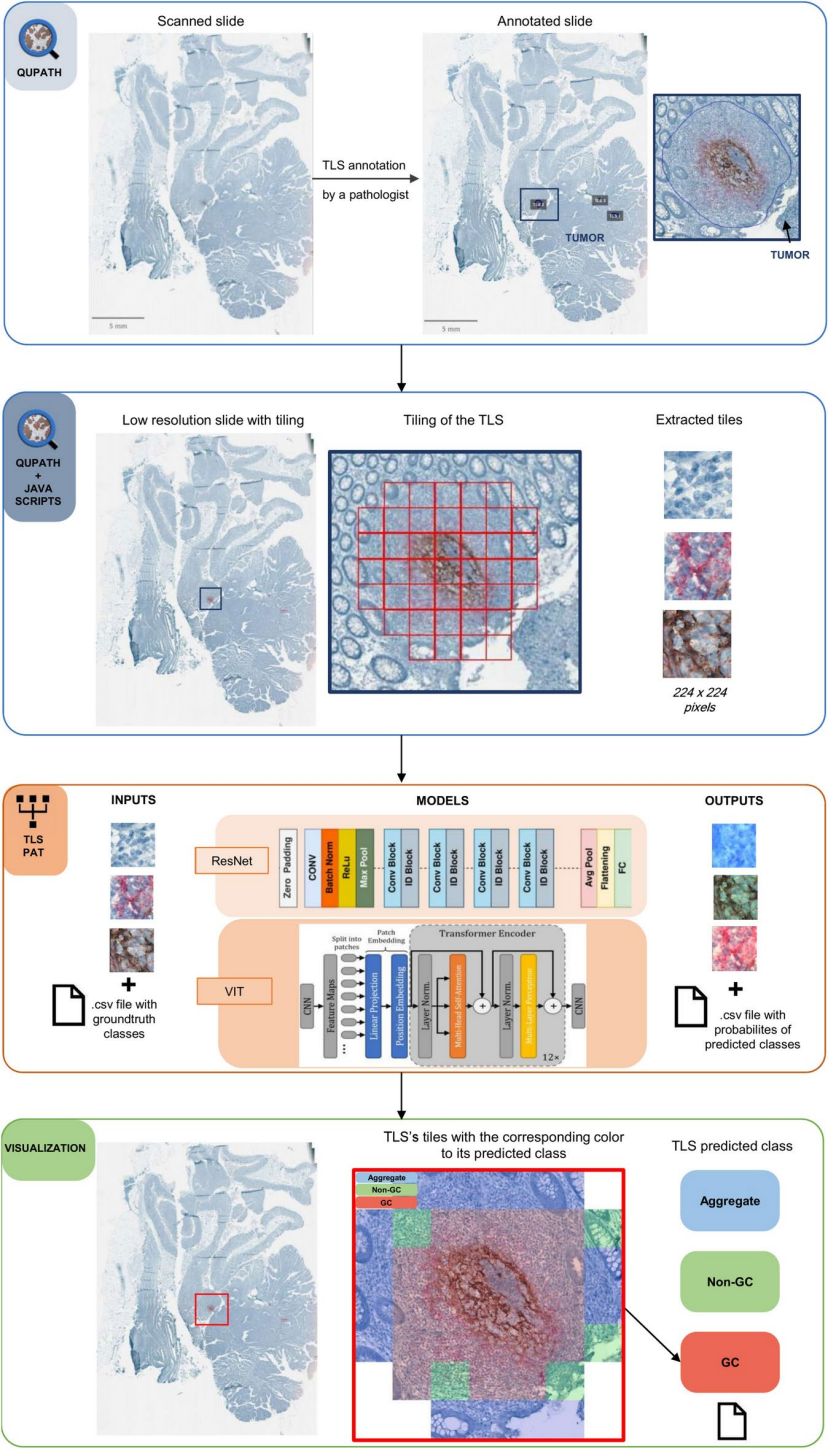
Novel Automated Pipeline for TLS Maturation Classification. First the slide is scanned and TLS are detected and annotated by a pathologist in QuPath. Secondly, using a java script, we generated a low-resolution image of the whole slide with its tiled regions, and we extracted the tiles of each TLS. These tiles and their corresponding maturation class (csv file) are used as inputs for the best combination evaluation (model, pretraining, aggregation). As outputs, we obtained the same tiles with a color filter corresponding to its predicted class (csv file): Blue for Aggregate, Green for Non-GC and Red for GC. Tiles are then rearranged to allow a visualization of the results. Each tile colored with its predicted class, and the predicted class of the whole TLS (csv file), depending on the aggregation method chosen.

The Test Set contained 133 patients and acted as the final evaluation metric, assessing how well the models perform on unseen data. The split was implemented to ensure that the different classes were proportionately represented in each set (Fig. 1E). Tiles were then extracted from the annotated TLS, to train and evaluate different models. The final objective was to have colored tiles as outputs in order to have visual results with colored tiles corresponding to their predicted class: Blue corresponding to class 1 Aggregate, Green corresponding to class 2 Non-GC, and red to class 3 GC-TLS. After a rearrangement of the tiles, we have a visual result of the predicted class of each tile and the whole TLS (Fig. 2).

### Cross validation

**Fig. 3:**
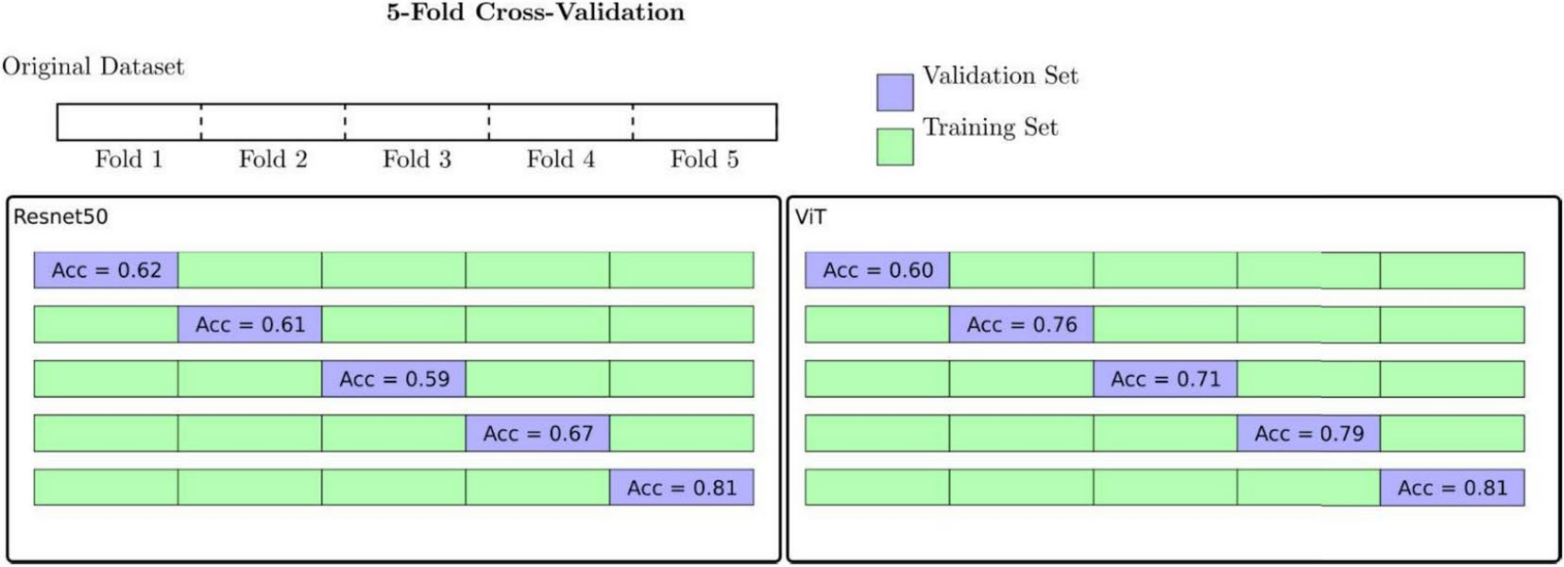
Performance Comparison of ResNet50 and Vision Transformer (ViT) Models Using 5-Fold Cross-Validation. On the left, the accuracy results of the 5-Fold Cross-Validation with the Resnet50 model. On the right, the accuracy results of the 5-Fold Cross-Validation with the ViT large model.

Cross-validation is a statistical method used to evaluate machine learning models by dividing the original dataset into a set of training and validation subsets, to ensure the model’s ability to generalize to an independent dataset. This technique is used to mitigate overfitting and to ensure that the model performs well on unseen data. The choice of 5 folds represents a balance between bias and variance. With fewer folds (e.g., 2 or 3), the training set is larger, but the validation set is smaller, leading to higher variance. We conducted a 5-Fold Cross-Validation using two distinct model architectures: Resnet50 and ViT large (Fig. 3). For Resnet50, the accuracy across Folds 1 to 5 ranged from 0.59 to 0.81. In contrast, the ViT large model demonstrated a slightly higher accuracy range, from 0.60 to 0.81. Notably, both models achieved their highest accuracy of 0.81 in Fold 5. Consequently, we selected Fold 5 for further analysis throughout this article.

### Aggregation methods

We evaluated various aggregation methods to identify the one that best fits our biological context and yields the highest accuracy in TLS classification:

1. *Majority Voting*: Aggregates predictions by selecting the class label that appears most frequently among the predictions for each image.
2. *Average Class*: Calculates the average of the predicted class labels across all images.
3. *Median*: Finds the median of the predicted class labels across all images.
4. *Mode*: Selects the most frequent class label among the predictions for all images.
5. *Soft Voting*: Averages the probability distributions of the predictions for all images.
6. *Custom Aggregation Method*: Prioritizes GC-TLS if present, followed by Non-GC TLS, and defaults to Aggregate if neither is present.
7. *Max Confidence*: Selects the class with the highest confidence score from the outputs for all images.

**Fig. 4:**
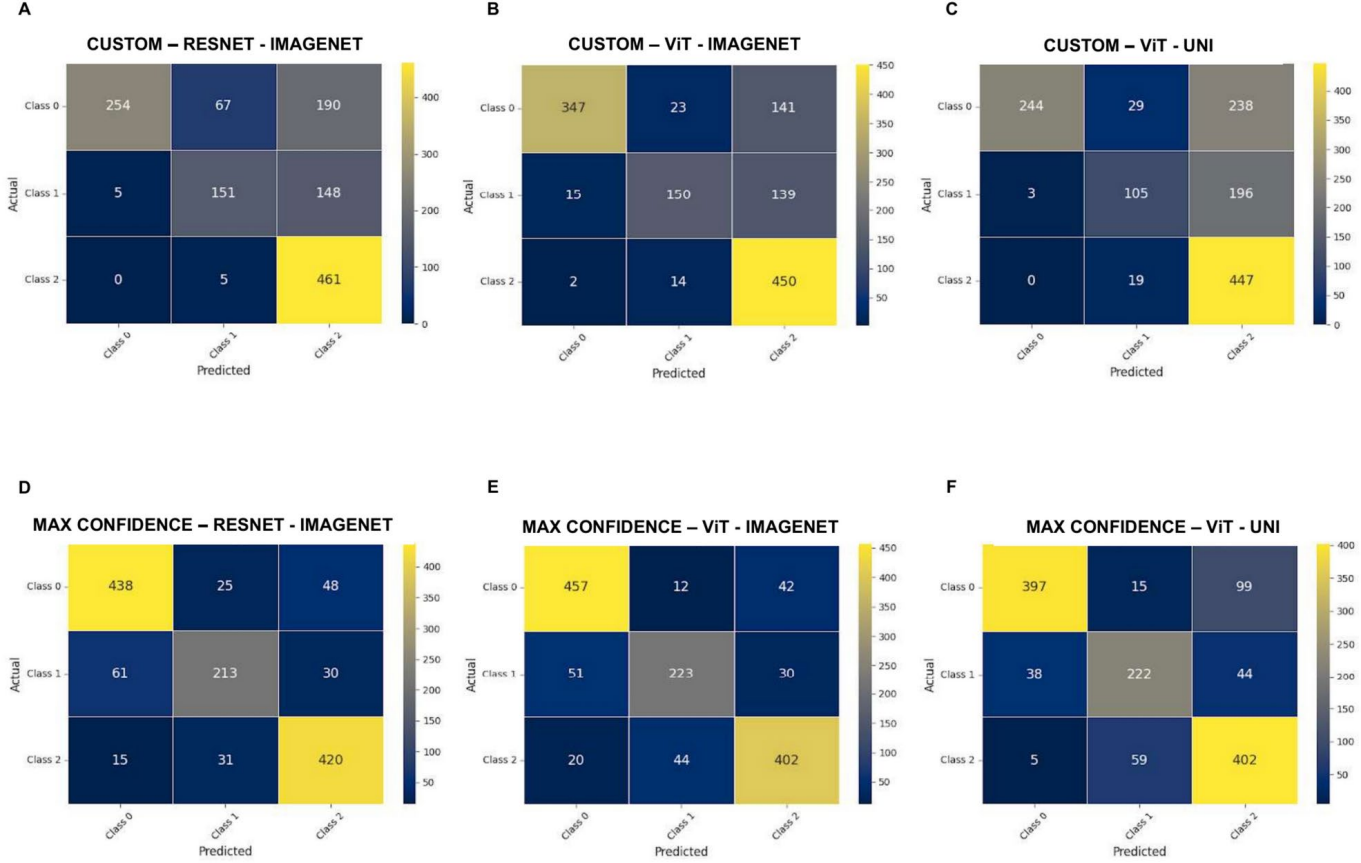
Normalized Confusion Matrices Comparing Custom and Max Confidence Aggregation Methods. The normalized confusion matrices using the Custom aggregation method with the Resnet-imagenet model (**A**), ViT-imagenet model (**B**) or the Vit-Uni model (**C**). The normalized confusion matrices using the Ax Confidence aggregation method with the Resnet-imagenet model (**D**), ViT-imagenet model (**E**) or the Vit-Uni model (**F**).

Accuracy of each Aggregation method is detailed in Supplementary Data 2, but we focused on the Custom Aggregation Method and the Max Confidence. Their performances were assessed using both raw (Supplementary Data 3) and normalized confusion matrices (Fig. 4), which illustrated the distribution of true positives (TP), false positives (FP), true negatives (TN), and false negatives (FN).

For the ResNet50 model pretrained on ImageNet with the Custom Aggregation Method, Class 1 (Aggregate) and Class 2 (Non-GC TLS) had low accuracies of 49.71% and 49.67%, respectively, while Class 3 (GC-TLS) achieved a high accuracy of 98.93% (Fig. 4A). The ViT large model with the same method also demonstrated variability: Aggregate had a correct prediction rate of 67.91%, Non-GC TLS at 49.34%, and GC TLS at 96.57% (Fig. 4B). In contrast, the ViT large model pretrained on UNI showed lower rates for Aggregate (47.75%) and Non-GC TLS (34.54%), but a high rate for GC TLS (95.92%) (Fig. 4C). However, this configuration led to 64.47% of Non-GC TLS being misclassified as GC-TLS.

The ResNet50 model using the Max Confidence method performed well, particularly for Class 1 and Class 3, achieving 70.07% accuracy for Non-GC TLS, with 20.07% misclassified as GC-TLS (Fig. 4D). The ViT large model pretrained on ImageNet with Max Confidence excelled overall, especially in distinguishing between Aggregate and GC TLS (Fig. 4E). In contrast, the ViT large model pretrained on UNI showed the lowest performance, with higher misclassification rates, particularly for Aggregate, indicating reduced effectiveness compared to the ImageNet-pretrained model (Fig. 4F).

### Model architectures

In this study, we compared two model architectures, ResNet50 and ViT large, using the Test set from Fold 5. We also evaluated different pretraining approaches: both models pretrained on ImageNet and ViT large trained exclusively on UNI. Table 1 summarizes the accuracy and Cohen’s kappa values based on these architectures and their aggregation methods.

**Table 1:** Accuracy of the Resnet50 and ViT models using different pretraining and Aggregation methods.

Model architecture	Pretrained	Aggregation method	Accuracy	Kappa
Resnet50	imagenet	Max Confidence	0.836	0.747
		Custom	0.676	0.502
ViT large	imagenet	Max Confidence	0.845	0.761
		Custom	0.739	0.595
ViT large	UNI	Max Confidence	0.797	0.689
		Custom	0.621	0.412

The ResNet50 model pretrained on ImageNet achieved a strong accuracy of 0.836 and a kappa of 0.747 using the Max Confidence method. However, performance dropped significantly with our Custom Aggregation Method, yielding an accuracy of 0.676 and a kappa of 0.502. In contrast, the ViT large model pretrained on ImageNet excelled with an accuracy of 0.845 and a kappa of 0.761 using the Max Confidence method. While it performed better than ResNet50 with the Custom Aggregation Method, its accuracy was 0.739 and kappa was 0.595. The ViT large model pretrained on UNI showed the lowest performance, with an accuracy of 0.797 and a kappa of 0.689 using the Max Confidence method, which further declined to 0.621 and 0.412 with the Custom Aggregation Method. Thus, the optimal combination for TLS classification was the ViT large model pretrained on ImageNet using the Max Confidence method.

To demonstrate the effectiveness of TLS-PAT, we compared its TLS classification performance against two trained pathologists (A.B. and M.C.) on a dataset of 70 new TLS images (Table 2). Inter-rater reliability, assessed using Cohen’s Kappa, showed substantial agreement between Rater 4 and Rater 5 (kappa = 0.7989) and between Rater 4 and TLS-PAT (kappa = 0.7149), while the agreement between Rater 5 and TLS-PAT was slightly lower (kappa = 0.6658). In terms of accuracy and classification time, Rater 4 achieved 90.0% accuracy in 9 min and 50 s, while Rater 5 had 85.7% accuracy in 9 min and 54 s (Table 3). In contrast, TLS-PAT demonstrated an accuracy of 80.0% on the new dataset, classifying all 70 TLS in just 15 s, with the calculation times being nearly identical and consistently fast across all models, showing no significant variations. TLS-PAT not only matched the raters’ agreement but also significantly outperformed them in classification speed, showcasing its efficiency and reliability.

**Table 2:** Table of the results of the Cohen’s Kappa test for inter-rater agreement between pathologists and TLS-PAT.

	Cohen’s Kappa [95% C.I.]	Level of inter-rater agreement
Rater 4 vs Rater 5	0.7419 [0.6122; 0.8716]	Substantial
Rater 4 vs TLS-PAT	0.7149 [0.5817; 0.8481]	Substantial
Rater 5 vs TLS-PAT	0.6658 [0.5095; 0.8221]	Moderate

**Table 3:** Table of the performances (accuracy and time for assessment) of pathologists and TLS-PAT.

	Accuracy	Time for classification
Rater 4	0.9000	9 min 50 s
Rater 5	0.8571	9 min 54 s
TLS-PAT	0.8000	15 s

### Data visualization

**Fig. 5:**
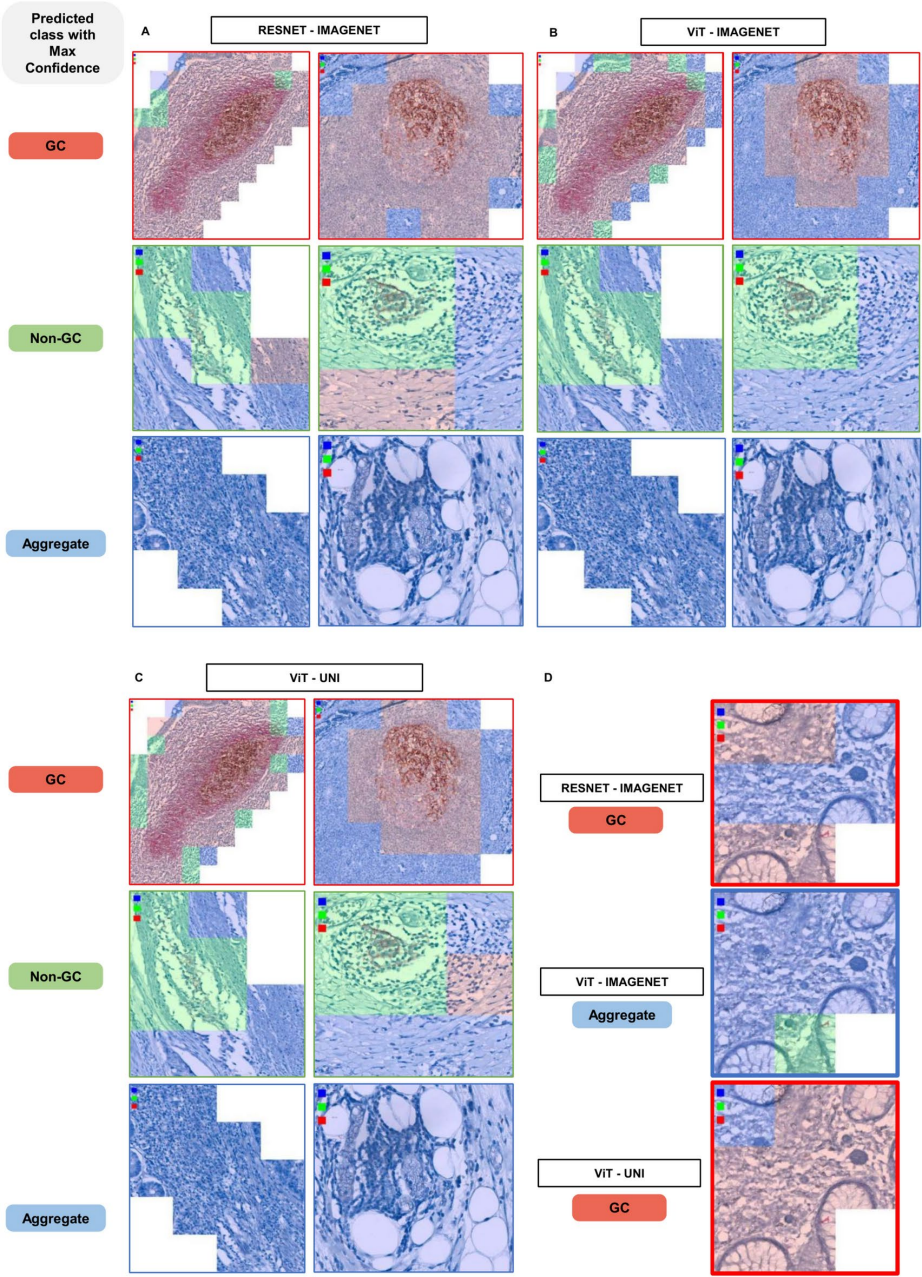
Visual Insights into Model Performance and Error Patterns. Colored tiles are rearranged to visualize each entire TLS. Here are examples for each TLS maturation class, with the Resnet-imagenet model (**A**), ViT-imagenet model (**B**) or the Vit-UNI model (**C**). We observed some misclassification with the Resnet-imagenet model and Vit-Uni model, compared to the ViT-imagenet model (**D**).

We aimed to visualize how each model classified the predicted classes on the test set. To achieve this, we generated colored tiles corresponding to their predicted classes: blue for class 1 (Aggregate), green for class 2 (Non-GC), and red for class 3 (GC-TLS). Rearranging these tiles provided a visual representation of the predicted class for each tile and the entire TLS (Fig. 5).

The output from the ResNet50-ImageNet model highlighted the distribution of predicted classes across different TLS regions, revealing some misclassifications, particularly between GC and Aggregate areas (Fig. 5A, D). In contrast, the ViT large model pre-trained on ImageNet demonstrated improved accuracy and fewer misclassifications, particularly in distinguishing GC from Aggregate regions (Fig. 5B, D). However, the ViT model pre-trained on UNI showed lower performance, with noticeable misclassifications between GC and Aggregate areas (Fig. 5C, D), indicating its reduced effectiveness when trained on this dataset.

Overall, these visualizations effectively illustrate the models’ performance in classifying TLS maturation stages. The ViT large model pre-trained on ImageNet yielded the most accurate and consistent results, with color-coded tiles providing a comprehensive view of spatial predictions and an intuitive assessment of each model’s classification capabilities.

## Discussion

In this study, we developed and validated an AI-based approach for the automated classification of tertiary lymphoid structures in colorectal cancer using dual immunohistochemistry. Our method, TLS-PAT, leveraging the ViT model, demonstrated high accuracy and robustness in classifying TLS maturation stages. This automated system not only streamlined the labor-intensive process of TLS evaluation but also proposed a possibility to harmonize the TLS assessment.

The accuracy (0.851) confidence matrix and Cohen’s kappa value (0.7961) indicate its reliability in distinguishing between TLS maturation stages, which is critical for prognosis and therapeutic decisions. Our results show significant potential for clinical applications, where the routine assessment of TLS could guide personalized treatment strategies in CRC patients. The ability to accurately classify TLS maturation stages using a simplified marker set (CD21, CD23) through this automated method provides a practical pathway for integration into clinical workflows.

In our study, we first evaluated various aggregation methods to identify the one that best fits our biological context and yields the highest accuracy in TLS classification. Despite Majority Voting, Average Class, Median, Mode and Soft Voting demonstrating good accuracy (Supplementary Data 2) (0.55–0.81), none of them fit perfectly with our specific biological context.

Thus, we created a custom aggregation method prioritizing classes based on their biological relevance, classifying even small positive cell clusters for one marker as indicative of the entire TLS. However, this method was sensitive to errors in tile predictions, affecting overall classification accuracy.

To overcome these limitations, we implemented the Max Confidence method, which selects the class with the highest confidence score from outputs. This method is well-suited for our needs, enhancing prediction accuracy and reliability by focusing on the most certain predictions.

Our evaluation of the ResNet50 and Vision Transformer (ViT) models revealed valuable insights. ResNet50 pretrained on ImageNet and using max confidence aggregation achieved an accuracy of 0.836 (kappa = 0.747). While effective with high-quality pretraining data, it struggled with custom aggregation, resulting in lower accuracy (0.676; kappa = 0.502).

ViT outperformed ResNet50 in all configurations, achieving the highest accuracy of 0.845 (kappa = 0.761) with ImageNet pretraining and max confidence aggregation. It remained robust even with custom aggregation (0.739; kappa = 0.595), although performance dipped with less optimal datasets. By allowing pretrained models to be adapted to domain-specific tasks, transfer learning has demonstrated impressive promise in medical image analysis. In order to improve feature extraction and diagnostic accuracy, Lu et al.^18^ used transfer learning in conjunction with ensembled randomized neural networks and featurenets to diagnose cerebral microbleeds. Transfer learning’s capacity to generalize across a variety of datasets was demonstrated by Zhang et al.^19^ when they used it to recognize food categories. Similar to this, Lu et al.^20^ demonstrated the benefits of pretrained models in high-dimensional medical picture datasets by using Vision Transformer (ViT) architectures for tuberculosis classification. Our use of ImageNet-pretrained ViT and ResNet50 models for TLS classification is based on the idea that transfer learning is essential for overcoming data scarcity and attaining excellent performance. These investigations demonstrate this point.

Key elements that maximize accuracy and robustness are responsible for our approach’s exceptional performance, TLS-PAT. A significant benefit of using the Vision Transformer (ViT) model was its capacity to collect global contextual information, which made it very useful for identifying intricate patterns in TLS development. The Max Confidence aggregation strategy, which reduced the influence of unclear tiles and concentrated on the most confident forecasts, improved classification reliability.

Additionally, compared to conventional H&E staining, the dual IHC markers (CD21/CD23) employed in our study offered greater specificity and sensitivity for TLS classification, guaranteeing accurate maturation stage identification. By carefully assessing performance across various data splits, extensive cross-validation using a fivefold technique greatly enhanced the model’s generalizability. These components work together to demonstrate how well TLS-PAT addresses the difficulties associated with automated TLS categorization in colorectal cancer.

Overall, the ViT model using ImageNet pretraining and max confidence aggregation emerged as the most effective configuration for TLS classification. This combination, dubbed TLS-PAT, demonstrated notable accuracy and efficiency, supporting its potential for clinical applications. Our approach offers significant advantages over existing models^8,13^ particularly through its use of dual IHC, which is more sensitive and specific for identifying TLS compared to H&E staining. This method accurately classifies double-positive GC-TLS, which are often misidentified in H&E-stained slides. Additionally, its integration with the QuPath interface enhances usability for pathologists, promoting rapid and consistent TLS analysis.

By minimizing inter-observer variability, our automated system ensures reproducible evaluations. The extensive cross-validation we conducted strengthened the model’s accuracy and robustness across different conditions. TLS-PAT empowers pathologists to define TLS in specific zones according to clinical needs, facilitating tailored analyses. Importantly, pathologists can visually assess the model’s predictions, enhancing trust and maintaining high diagnostic standards.

In summary, our method combines the strengths of dual IHC, robust model optimization, and seamless clinical integration, providing a powerful tool for TLS classification in both research and clinical settings.

Our study had limitations, including reliance on a single dataset from one center, emphasizing the need for further validation with external datasets to ensure generalizability. Testing across different cancer types and tissues would broaden the applicability of our approach.

The observed data imbalance, particularly the prevalence of GC TLS tiles, stems from their larger size compared to Aggregates and Non-GC TLS. To improve model performance, future work may incorporate data augmentation, oversampling underrepresented classes, or class weighting for a more balanced dataset.

Both our TLS-PAT tool and QuPath are open-source, allowing the research community to modify and enhance them. Future research will focus on automating TLS detection across all maturation stages, building upon existing work with H&E slides^8,13^. Particular emphasis will be placed on enhancing the identification of challenging “Aggregates” or double-negative TLS, and improving TLS detection in specific tumor zones on IHC slides.

Our goal is to offer a flexible tool that can integrate into existing workflows without replicating other teams’ efforts in TLS detection on H&E slides. Instead, we aim to enhance the accuracy of TLS maturation classification through IHC, as our dataset emphasizes TLS located within or near tumors, aligning with their biological definition.

In conclusion, this AI-driven approach represents a significant advancement in the automated analysis of TLS in colorectal cancer. With further validation and expansion, this methodology could play a critical role in routine clinical practice.

## Methods

### Patients and clinical data

All tissue samples for this study were sourced from the Department of Pathology at CHU Brest, France, following institutional review board approval (CHRU Brest, CPP n° AC-2019–3642) and in compliance with the Helsinki Declaration. The study involved 656 colorectal adenocarcinoma patients who underwent surgery between 2005 and 2021. Inclusion criteria were: (1) confirmed diagnosis of primary colon adenocarcinoma via pathological examination, (2) no prior preoperative chemotherapy or radiotherapy, (3) adequate formalin-fixed paraffin-embedded (FFPE) tissue specimens, (4) at least one slide containing the tumor’s invasive margin, and (5) at least one tertiary lymphoid structure (TLS) present in the tissue. An additional cohort of 13 patients meeting the same criteria was selected from late 2022 for the final dataset compilation (see Dataset section below).

### Sample selection and processing

All hematoxylin–eosin saffron (HES)-stained sections were reviewed by a pathologist to select the most appropriate formalin-fixed paraffin-embedded (FFPE) blocks, which included tumor tissue, the tumor front, and adjacent normal tissue. A total of 656 blocks were selected.

For each selected FFPE sample, two serial tissue sections were cut at 3 μm and mounted on Superfrost® Plus slides, dried overnight at 37 °C, and processed for immunohistochemistry (IHC) using the Ventana Benchmark Ultra® system with the ultraView Universal DAB and Alkaline Phosphatase Red Detection Kits. Each marker underwent a pretreatment with cell conditioner 1 (CC1 pH 8) for either 36 or 64 min, followed by antibody incubation at 37 °C for 20 or 32 min. The antibodies used for IHC included: Slide 1: anti-CD20 (clone L26, 1:100 dilution, 20 min, CC1 36 min) in red, anti-CD3 (polyclonal, 1:100, 32 min, CC1 36 min) in DAB. Slide 2: anti-CD21 (clone 2G9, 1:50, 32 min, CC1 64 min) in red, anti-CD23 (1:50, 32 min, CC1 64 min) in DAB. After washing, the slides were counterstained with hematoxylin for 12 min and bluing reagent for 4 min, washed in water with dishwashing detergent, and coverslipped.

### Pathological image analysis

The first step was the brightfield slides digitalization, histochemical stained slides and immunohistochemistry slides were scanned at 20X (0.5mpp) in a mrxs format using a 3DHistech Panoramic Midi slide scanner (3DHISTECH, Budapest, Hungary).

To evaluate our maturation annotation method, three different raters (M.G, P.L.N, M.L.R) underwent independent training using a dataset comprising 9 typical cases, encompassing samples with no TLSs, aggregates, Non-GC TLS and GC TLS. The interrater agreement score for TLS presence was evaluated by a Cohen’s kappa test. A kappa score lower than 0.20 suggests minimal agreement, while a score between 0.21 and 0.40 indicates fair agreement. Moderate agreement falls within the range of 0.41 to 0.60, substantial agreement from 0.61 to 0.80, and almost perfect agreement between 0.81 and 1^14,15^.

QuPath software v0.5.0 was then used for the annotations of the 656 digital slides by a pathologist for the quantitative image analysis of TLS (annotated by a single polygon per TLS). Manual annotation was necessary to create a reliable ground truth dataset. In our assessment, TLS were defined as clusters or aggregates of B cells (CD20 +) and T cells (CD3 +), readily identifiable on slide 1 within the tumor or at the invasive margin (1 mm from the closest cancer cell), excluding the secondary lymphoid organs. Using the slide 2, TLS maturation groups are defined as Aggregates when the TLS is CD21- and CD23-, Non-GC TLS when the TLS is CD21 + and CD23-, and as GC-like TLS when the TLS is CD21 + and CD23 + . For the 656 patients of the cohort, a total of 6353 TLS was exhaustively annotated. To perform patch-based analysis, non-overlapped tiles 224 × 224 pixels were extracted from the annotated “TLS”, using the “Tiles_extract_TLS” script (soon available on Github: https://github.com/LBAI-InfoLab). A low-resolution image of the tissue is generated for each slide to see the location of each tile extracted. A total of 100 245 tiles have been exported from QuPath.

### Dataset

The dataset included a diverse range of TLS localizations, sizes, forms, and maturation stages, providing a comprehensive representation of TLS complexities. Additionally, the dataset encompassed tissues of varying ages, resulting in different staining intensities and artifacts. The dataset was divided into training, validation, and test sets using DataFrames, each of which included information about the specific split type (i.e., training, validation, or test). This helps make sure that the models are evaluated fairly and that the results are generalizable to a broader population. A final dataset of 70 new TLS images (from 13 new patients of 2022 chosen with the same inclusion criteria as previously described) was compiled to compare the classification performance between pathologists (Rater 4 A.B and Rater 5 M.C) and AI.

### Model architecture

#### Resnet description

ResNet, introduced by He et al.^16^, addresses the vanishing gradient problem encountered in training very deep networks. In this study, ResNet was selected for its strong performance in image classification and ability to extract hierarchical features from complex images like Whole Slide Images (WSIs) of Tertiary Lymphoid Structures (TLS).

#### ViT description

Vision Transformer (ViT), proposed by Dosovitskiy et al.^17^, applies the transformer architecture to image classification by treating images as sequences of patches. ViT has demonstrated competitive performance by effectively capturing global dependencies, making it particularly suitable for tasks such as in analyzing TLS variations in histopathology. Here, we used the ViT large model, which offers increased depth and is more suitable for high-performance tasks.

#### Overall training pipeline

The overall training pipeline involved several key steps to effectively utilize ResNet and ViT architectures on the TLS dataset. (1) Data Preprocessing, tiles of size 224 × 224 pixels were extracted from annotated TLS using the “Tiles_extract_TLS” script. A low-resolution image of tissue was generated for each slide to visualize tile locations. (2) Model Initialization, ResNet and ViT models were initialized with pretrained weights from large-scale image datasets like ImageNet and UNI, a specialized dataset designed for histopathology images. This initialization helped bootstrap model learning and accelerate convergence by providing a solid foundation of feature representations tailored for both general and domain-specific image characteristics. (3) Validation and Fine-tuning, model performance was evaluated on the validation set to monitor metrics such as accuracy, precision, and recall. Hyperparameters were adjusted based on validation results to improve model performance and prevent overfitting. (4) Testing and Evaluation, finally, the trained models were evaluated on the test set to assess their generalization capability and effectiveness in classifying TLS in unseen data.

### General training setup summary

The training framework for our study was implemented using the PyTorch library, which provides a robust platform for developing deep learning models. Data loading was handled efficiently using Pandas, ensuring that the data was properly structured and split into training, validation, and test sets. This division was crucial for evaluating the model’s performance and preventing overfitting. To ensure reproducibility across different runs of the experiment, specific seeds were set, allowing for consistent results and facilitating the verification of findings.

For model selection, we employed a range of pre-trained models, including ResNet and ViT large architectures using the Timm library. These models were chosen for their proven performance in various computer vision tasks. Additionally, custom classifiers were integrated into the architecture to tailor the models to our specific classification problem. The primary loss function used was Cross-Entropy with Logits Loss, which is well-suited for multi-class classification problems.

Optimization of the model was performed using specific optimization methods, with the learning rate being dynamically adjusted using a StepLR scheduler. This approach helps in fine-tuning the learning process, preventing the model from converging too quickly or getting stuck in local minima. Model evaluation was performed using various metrics, including: Accuracy, Confusion Matrix and Cohen’s Kappa.

### Workstation configuration

The workstation used for this study includes NVIDIA RTX A6000 with 48 GB of VRAM with an operating system Ubuntu 20.04 LTS. It has 128 GB of DDR4 RAM, ensuring ample memory for handling large datasets and complex computations. We used Python (3.8.10) as programming language with the principle libraries: PyTorch Version: 1.10.2, Torchvision Version: 0.11.3,Timm Version: 0.4.12,Pandas Version: 1.1.5,JupyterLab Version: 3.2.9. More details and the code are available at https://github.com/LBAI-InfoLab.

## Supplementary Information

Supplementary Information 1.

Supplementary Information 2.

Supplementary Information 3.

## Data Availability

All the codes used for this project will be available on GitHub: https://github.com/LBAI-InfoLab.

## Abbreviations

CRC: Colorectal cancer
IHC: Immunohistochemistry
TME: Tumor microenvironment
HE: Hematoxylin-eosin
TLS: Tertiary Lymphoid Structures
WSI: Whole-slide images
