# Supplementary material for: Automated classification of tertiary lymphoid structures in colorectal cancer using TLS-PAT artificial intelligence tool
Source: Sci Rep. 2025 Mar 21;15:9845. doi: 10.1038/s41598-025-94664-0 (PMC11928541; doi:10.1038/s41598-025-94664-0)
Supplement: Supplementary file 1 — Supplementary Information 1. [file 41598_2025_94664_MOESM1_ESM.pdf]

Supplementary Data 1: Table of the results of the Cohen's Kappa test for interrater agreement assessment for TLS maturation evaluation.

	Cohen's Kappa [95% C.I.]	Level of inter-rater agreement
Rater 1 vs Rater 2	0,80057 [0,66265 ;0,93849]	Strong
Rater 2 vs Rater 3	0,85896 [0,74115 ;0,97676]	Strong
Rater 1 vs Rater 3	0,88522 [0,77609 ;0,99435]	Strong
