# Supplementary material for: Automated classification of tertiary lymphoid structures in colorectal cancer using TLS-PAT artificial intelligence tool
Source: Sci Rep. 2025 Mar 21;15:9845. doi: 10.1038/s41598-025-94664-0 (PMC11928541; doi:10.1038/s41598-025-94664-0)
Supplement: Supplementary file 2 — Supplementary Information 2. [file 41598_2025_94664_MOESM2_ESM.pdf]

Supplementary Data 2 : Aggregation methods performances using Fold 5.

Model	Aggregation Method	Accuracy	Kappa
ViT- UNI	Max confidence	0.7970335675253708	0.6893580944013387
	Custom	0.6213895394223263	0.4125997696858992
	Soft voting	0.7478532396565184	0.607554632369679
	Mode	0.6307572209211554	0.4152564700160104
	Majority	0.6307572209211554	0.4152564700160104
	Median	0.6174863387978142	0.40448168588351485
	Average class	0.550351288056206	0.3320115807255879
RESNET50 - imagenet	Max confidence	0.8360655737704918	0.7472810233892583
	Custom	0.6760343481654957	0.5024595549216369
	Soft voting	0.8157689305230289	0.7137752681972205
	Mode	0.7782982045277127	0.6535437569995199
	Majority	0.7782982045277127	0.6535437569995199
	Median	0.7619047619047619	0.6318601331392308
	Average class	0.6947697111631538	0.5423202390416404
VIT - imagenet	Max confidence	0.8446526151444185	0.760741205853285
	Custom	0.7392661982825918	0.5955739940960121
	Soft voting	0.7494145199063232	0.6054513215237678
	Mode	0.6494925839188135	0.4408125765618619
	Majority	0.6494925839188135	0.4408125765618619
	Median	0.6135831381733021	0.39012496717844247
	Average class	0.5511319281811085	0.32153486071465087
