# Supplementary figures and images for: Automated classification of tertiary lymphoid structures in colorectal cancer using TLS-PAT artificial intelligence tool
Source: Sci Rep. 2025 Mar 21;15:9845. doi: 10.1038/s41598-025-94664-0 (PMC11928541; doi:10.1038/s41598-025-94664-0)

A

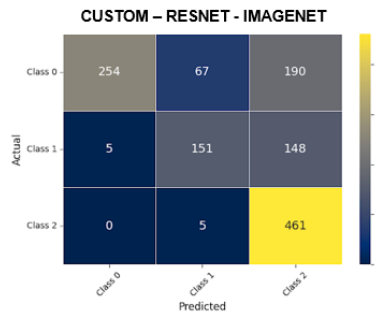

B

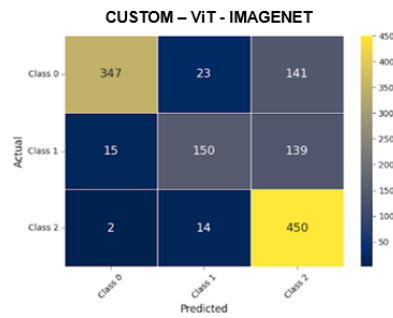

C

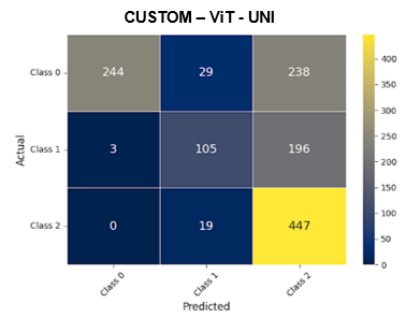

D

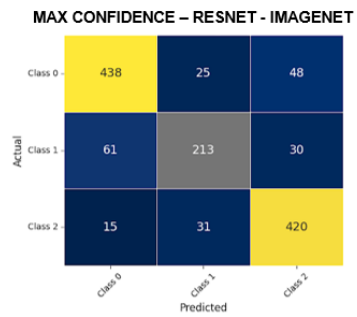

E

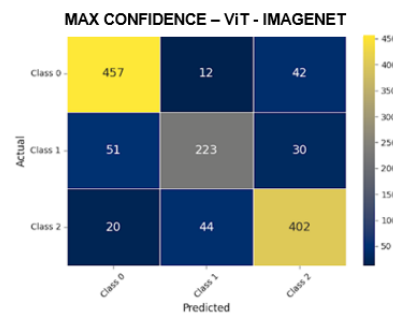

F

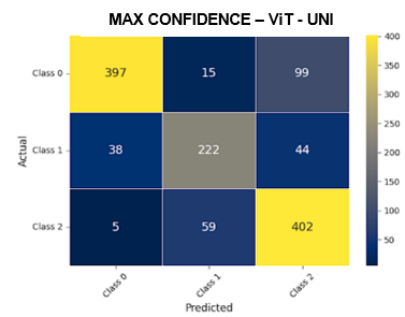

Supplement: Supplementary file 3 — Supplementary Information 3. [file 41598_2025_94664_MOESM3_ESM.pdf]
